# Posterior Reversible Leukoencephalopathy Syndrome in a Pre-Eclamptic Woman

**DOI:** 10.1155/2013/783536

**Published:** 2013-02-20

**Authors:** Raghvendra Thakur, Bhoj Raj Sharma, Tian Yuan, Zheng Guiying

**Affiliations:** ^1^Department of Obstetrics and Gynecology, Second Hospital of Jilin University, Norman Bethune College of Medicine, Ziqiang Street, Changchun 130041, China; ^2^Department of Diagnostic Radiology, Second Hospital of Jilin University, Norman Bethune College of Medicine, Ziqiang Street, Changchun 130041, China

## Abstract

Posterior reversible leukoencephalopathy syndrome (PRLS) is a neurologic condition, often presents with various clinical manifestations. It is rare during pregnancy. We herein report a case of PRLS in a 31-year-old woman, presented with preeclampsia, diagnosed by computer tomography, and successfully delivered the baby with caesarean section. At discharge, patient was fully recovered without any complication.

## 1. Introduction

Reversible Posterior Leukoencephalopathy Syndrome (RPLS) was first described by Hinchey et al. in 1996 [1]. It is a rare and a serious syndrome of central nervous system that can develop in both adults and children. It can be diagnosed with clinical presentation and imaging studies. Here, we present a case of PRLS in a 31-year-old female, with 36 weeks of gestation. The following case shows the importance of prompt diagnosis by computed tomography (CT) and management.

## 2. Case Report

**Figure 1:**
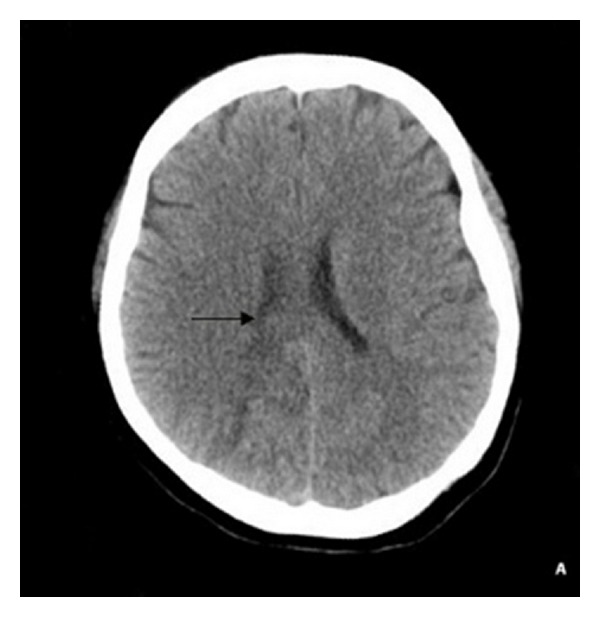
Unenhanced axial CT scan of the head shows a hypodense area (arrow) in the white matter of right parietal lobe, extending forward to right lateral ventricle with no midline shift.

A 31-year-old primigravida in her 36 weeks of gestation came to our hospital with sudden dizziness, three episodes of seizure within 20 minutes followed by unconsciousness. Seizure was generalized tonic-clonic seizure (GTCS) type and each lasted for approximately 15 second. She was known case of gestational hypertension under medication of tablet Labetalol 5 mg daily. At presentation her blood pressure was 170/110 mm of Hg pulse rate 138/minute, bilateral pedal oedema with fetal heart sound 142/min. Immediately Injection Diazepam 10 mg and Magnesium sulphate 5 gm. Bolus dose followed by 2 gm. Over 40 minutes in i/v infusion was given. Patient was admitted and shifted to obstetrics Intensive Care Unit (ICU) with diagnosis of eclampsia and differential diagnosis of intracranial hemorrhage, PRES. Biochemical and laboratory tests were unremarkable except with 3+ proteinuria on urine deep stick test. Patient had recurrent seizure in ICU so, and urgent CT was advised. CT head showed low density shadow on bilateral parietal lobe, high density shadow near the top of the cerebral sulcus (shown in Figure 1). So with respect to the CT findings and clinical presentation, RPLS was diagnosed and prompt caesarian section was done without any complication. Post operatively patient had no episodes of seizures and blood pressure was controlled with medication. Repeat CT scan of head on 7th day of postoperative showed disappearance of low density and high density shadows. She was discharged after one week, with oral antihypertensive medication, and patient had no recurrences of symptoms after 2 months of discharge.

## 3. Discussion

Narbone et al. proposed the term posterior reversible encephalopathy syndrome (PRLS) in 2006 [2]. Many causes have been postulated, such as pre-eclampsia, eclampsia, cerebral infarction, intracerebral hemorrhage, hypertensive encephalopathy, and autoimmune disease [1, 3]. However, underlying pathology still remains unclear. Two theories were suggested by Bartynski. First, hyperperfusion related to hypertension; after exposure to causative agent, autoregulation mechanism of intracranial pressure fails, leading to endothelial injury and vasogenic edema (as seen on CT of this patient), subsequently causing vasoconstriction and hypoperfusion, leading to vasogenic brain edema and ischemia [3]. Clinical features are typically characterized by headache, vomiting, visual disturbances, confusion, altered sensorium, and seizure, which is usually tonic-clonic [4]. Our patient did not have any focal deficits or visual abnormalities which was proven by optical coherence tomography test. However, our patient had multiple episodes of GTCS with severe hypertension, which precipitated PRLE. CT shows bilateral symmetrical hypodensities in the brain especially white matter, sometimes in grey matter. These lesions are common in parietal and occipital region of brain. MRI hypointense to isointense in T1W1 and hyperintense in T2W2, but hypointense or isointense in diffusion weighted imaging [5, 6]. Although MRI yields a higher resolution image, Hinchey et al. considered CT rather than MR for diagnosis of disease [1]. Control of blood pressure is vital to prevent irreversible damage to brain, such as chronic epilepsy or death [7].

## 4. Conclusion

PRES is difficult to diagnose and treat when it develops in eclampsia and pre-eclampsia so, as to prevent neurological consequences. Although we were in dilemma whether to do CT or MRI in pregnant woman because CT has radiation hazard, we chose CT as it is a rapid method of diagnosis in this critical condition. However MRI is safe in pregnancy, and diagnostic as CT, because of its prolonged scanned time with its heavy noise, compelling us to choose CT as a diagnostic modality. Our case illustrates that patient with repeated seizure can be successfully diagnosed PRES with CT.
